# Supplementary material for: Using remarkability to define coastal flooding thresholds
Source: Nat Commun. 2020 Feb 4;11:530. doi: 10.1038/s41467-019-13935-3 (PMC7000679; doi:10.1038/s41467-019-13935-3)
Supplement: Supplementary file 1 — Supplementary Information [file 41467_2019_13935_MOESM1_ESM.pdf]

Using Remarkability to Define Coastal Flooding Thresholds

Supplementary Information

Moore and Obradovich

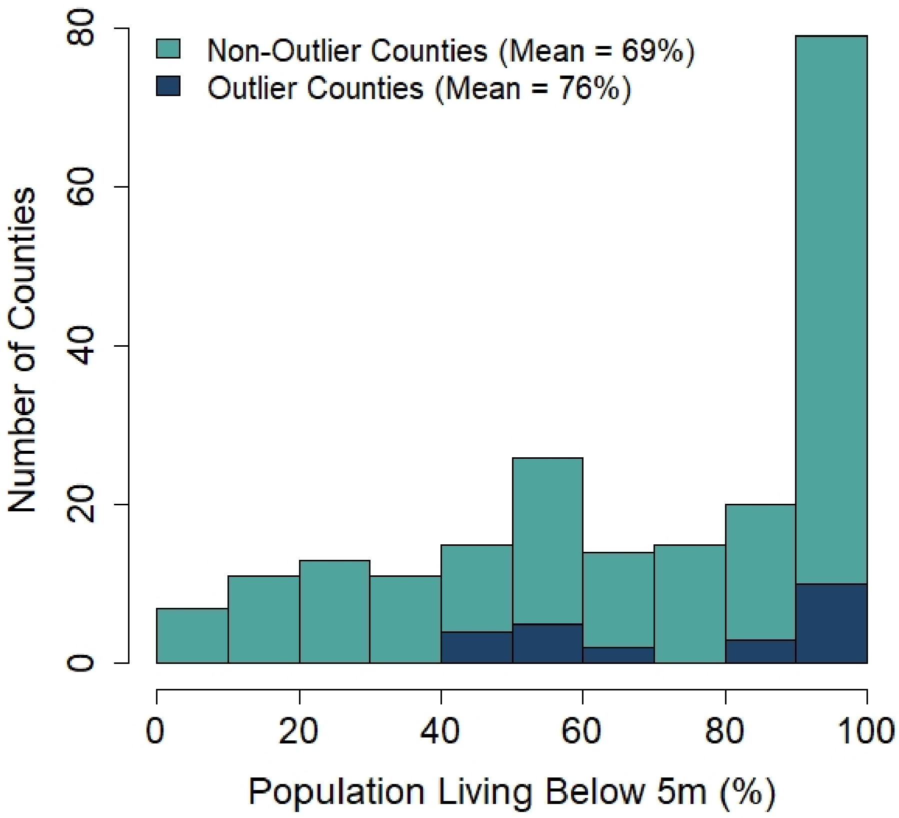

Supplementary Figure 1: Distribution of county population at low elevation for the 18 counties identified as having noticeable flood thresholds that differ significantly from the minor flooding threshold of the nearest tide gauge

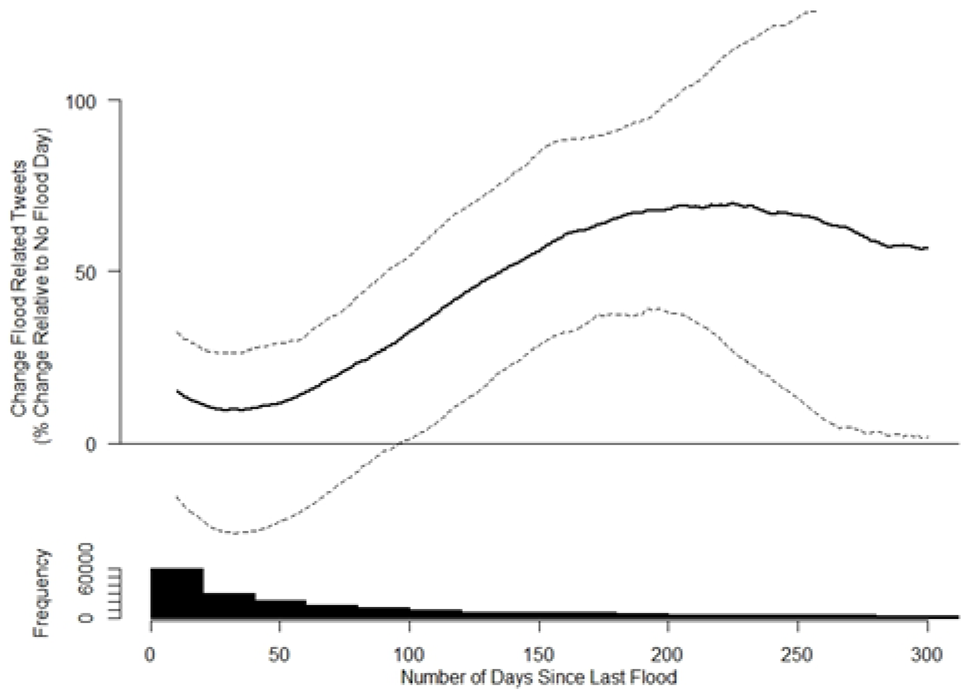

Supplementary Figure 2: Relationship between frequency of flooding and number of flood tweets. Response function showing % change in the number of flood-related Tweets over the whole sample on flood days, as a function of number of days since the last flood event (upper panel). Regression is a negative binomial regression at the daily level and includes controls for daily precipitation (quartic), cumulative 5 day precipitation (quadratic), the number of Twitter users, as well as county, state-month, and year fixed effects and county-specific time trends. There is evidence that the Twitter response to flooding is larger if the last flood was several months ago compared to very recently. Since much of the variation used to estimate the response function comes from difference in baseline flood frequency between counties, whether this response represents a normalization effect (flooding experienced more often becomes less remarkable) or an adaptation effect (counties that experience regular flooding at adapted to make the flooding less consequential) is unclear. Lower panel shows the frequency of intervals between floods.

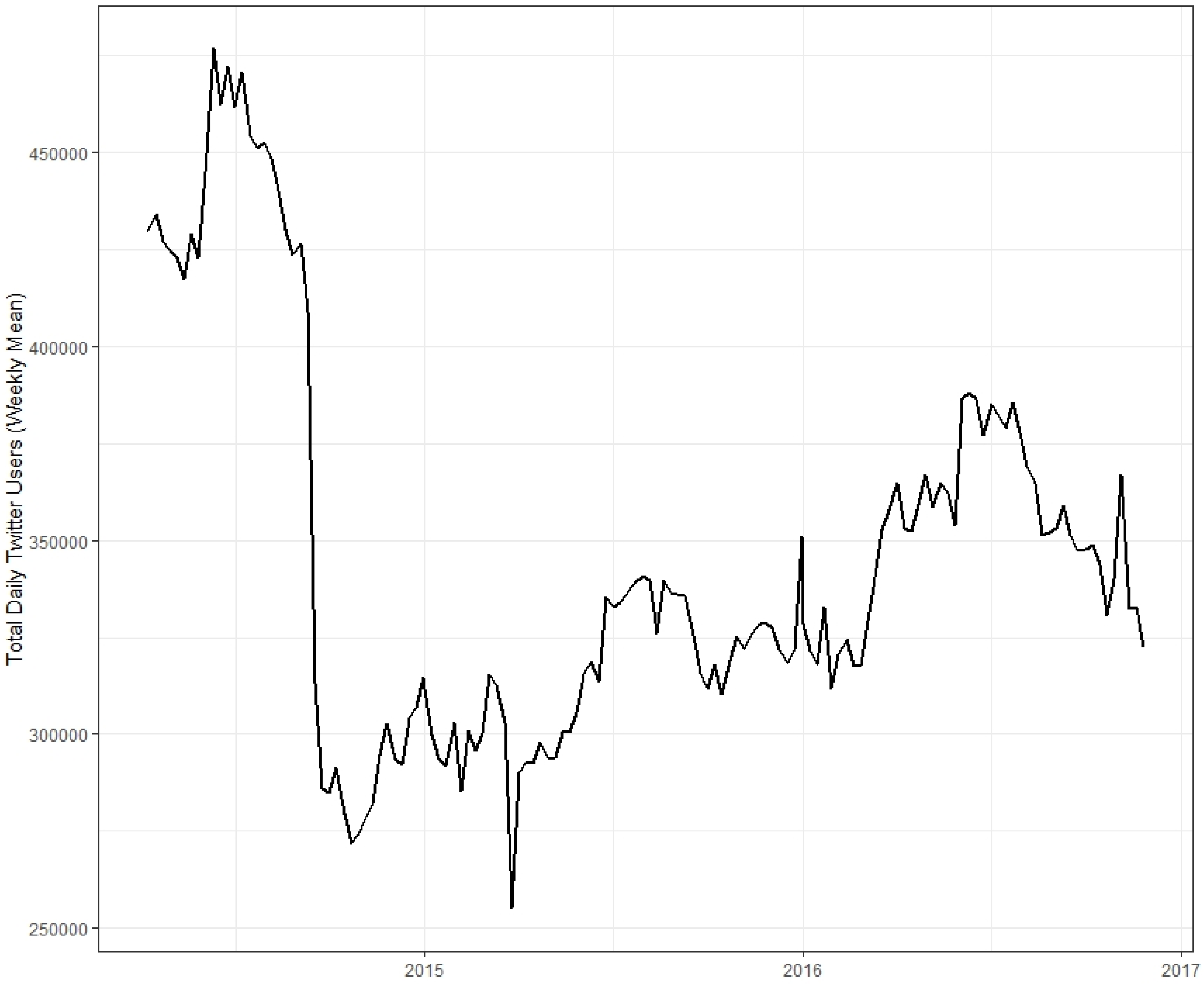

Supplementary Figure 3: Average daily unique Twitter users in the sample of shoreline counties by week. There is a general increasing trend interrupted by a sharp drop in late 2014 likely associated with a change in Twitter's opt-in policy on geolocating Tweets.

State	Flood HighLow	Name	Notes	Cluster	Tide Gauge	Distance to Gauge
CT	High	New Haven County	Long Island Sound	New York and Long Island Sound	New Haven	In county
FL	High	Bay County	Middle of Florida Panhandle,		Panama City	In county
FL	High	Duval County	Jacksonville		Mayport	In county
FL	High	Miami-Dade County	Miami, south eastern tip of FL		Virginia Key Biscayne Bay	In county
ME	High	Hancock County	Near Bangor		Bar Harbour	In county
MA	High	Barnstable County	Cape Cod	Cape Cod, Boston, Rhode Island	Woods Hole	In county
MA	High	Bristol County	East of Providence and west of Cape Cod	Cape Cod, Boston, Rhode Island	Fall River	In county
MA	High	Essex County	North of Boston	Cape Cod, Boston, Rhode Island	Boston	Neighboring County
MA	High	Plymouth County	Between Boston and Cape Cod	Cape Cod, Boston, Rhode Island	Boston	40 miles
NJ	High	Atlantic County	Atlantic City	Southern New Jersey	Atlantic City	In county
NJ	High	Camden County	Adjacent to Atlantic County	Southern New Jersey	Philadelphia	Neighboring County
NJ	High	Cumberland County	Adjacent to Atlantic County on Delaware Bay	Southern New Jersey	Ship John Shoal	In county
NJ	High	Hudson County	Adjacent to New York City on Lower Hudson River	New York and Long Island Sound	New York City	Neighboring County
NY	High	New York County	Manhattan	New York and Long Island Sound	New York City	In county
NY	High	Suffolk County	End of Long Island	New York and Long Island Sound	Bridgeport	45 miles
RI	High	Kent County	South of Providence	Cape Cod, Boston, Rhode Island	Quonset Point	In county
TX	High	Cameron County	Brownsville	Texas Coast	Port Isabel	In county
TX	High	Galveston County	Galveston	Texas Coast	Eagle Point	In county
TX	High	Jefferson County	Beaumont and Port Arthur east of Houston	Texas Coast	Sabine Pass North	In county
TX	High	Nueces County	Corpus Christi	Texas Coast	Corpus Christi	In county
TX	High	Orange County	Just east of Beaumont (neighbors Jefferson)	Texas Coast	Sabine Pass North	Neighboring County
VA	High	Cheasapeake City	South of Norfolk and Virginia Beach		Money Point	In county
NJ	Low	Cape May County	Cape May		Cape May	In county
NC	Low	New Hanover County	Wilmington NC adjacent to Brunswick County		Wilmington	In county

Supplementary Table 1: Outlier Counties. Details on counties with noticeable flooding thresholds that differ significantly from the minor flooding threshold of the nearest tide gauge. Outlier type indicates whether the minor flooding threshold is higher (high) or lower (low) than the estimated noticeable flood threshold. Cluster column indicates neighboring counties that are both identified as outliers. Green identifies counties where the matched tide gauge is either in that county or in a neighboring county.

a) Dependent Variable: Absolute value of difference between gauge and estimated flood thresholds as quantiles of tide height distribution

	**Model 1**	**Model 2**
log(mean number Twitter users)	-1.069<br>(0.735)	-0.984<br>(0.774)
log(distance to nearest tide gauge)	--	0.442<br>(1.224)

b) Dependent Variable: Difference between gauge and estimated flood threshold, as quantiles of tide height distribution

	**Model 1**	**Model 2**
log(mean number Twitter users)	-0.667<br>(0.8212)	-0.534<br>(0.864)
log(distance to nearest tide gauge)	--	0.695<br>(1.367)

c) Dependent Variable: Standard error of noticeable flooding threshold

	**Model 1**	**Model 2**
log(mean number Twitter users)	-0.835*<br>(0.337)	-0.720*<br>(0.350)
log(distance to nearest tide gauge)	--	0.634<br>(0.537)

Code: * $p<0.05$, ** $p<0.01$, *** $p<0.001$

Supplementary Table 2: Population Density and Estimated Flood Thresholds. a) Two models regressing the absolute difference between gauge and estimated flood thresholds on the number of Twitter users in a county (Model 1) and both the number of Twitter users and the distance to the nearest tide gauge (Model 2). b) Two models regressing the difference between gauge and estimated flood thresholds on the number of Twitter users in a county (Model 1) and both the number of Twitter users and the distance to the nearest tide gauge (Model 2). c) Two models regressing the difference between gauge and estimated flood thresholds on the number of Twitter users in a county (Model 1) and both the number of Twitter users and the distance to the nearest tide gauge (Model 2). Numbers in parentheses show the standard error of the estimate.

There is no evidence of a relationship between either the absolute value or difference between gauge and estimated flood thresholds and the Twitter population of a county. Results are shown with and without controls for distance to nearest tide gauge, which might be expected to correlate both with Twitter population (i.e. more rural areas have fewer Twitter users and are farther from tide gauges) and the estimated differences in flood thresholds. Consistent with the number of Twitter users in a county increasing the variance of estimates, we find a statistically significant relationship between the size of the Twitter population and the standard error of the noticeable flooding threshold (Table c). This suggests that the number of Twitter users affects the variance of estimated thresholds but does not bias estimates in either direction.

	Flood-Stage Coefficient	Standard Error	p-value
True-Positive Flood Tweets	1.254	0.193	<0.0001
False-Positive Flood Tweets	0.024	0.937	0.980

Supplementary Table 3: Results of Manual Validation of Flood Tweet Classification. Coefficient on an indicator variable for whether daily tide height was above gauge flood threshold with two dependent variables in two negative binomial regressions. Top row is the number of tweets about flooding identified as true positives, bottom row is the number of false positive tweets about flooding. Both regressions include controls for daily precipitation (quartic), five day precipitation (quadratic), number of Twitter users and month-of-year and county fixed effects. There is no evidence that the number of false-positive tweets are associated with extreme tide heights (bottom row), meaning error from the high rate of false-positives (64%) will introduce noise but not bias into our estimates. Data is from 3305 manually-validated tweets from three counties randomly-selected from the set of all outlier counties.

Note that this analysis focuses on false-positive errors, which are a far larger concern than false-negatives simply because tweets about flooding are an extremely small fraction of overall tweets (less than 0.05% of all geolocated Tweets in our dataset). In addition, the bag-of-words (Methods) is designed to be large, so as to generally generate more false positive than false negatives. Given these priors, the false-negative rate should be extremely low. This was found in a previous validation of classification of tweets about weather using the same bag-of-words approach reported in Moore et al. (2019) - the false-positive rate was ~ 45% while the false negative rate was <0.5%.
