# Supplementary material for: Using remarkability to define coastal flooding thresholds
Source: Nat Commun. 2020 Feb 4;11:530. doi: 10.1038/s41467-019-13935-3 (PMC7000679; doi:10.1038/s41467-019-13935-3)
Supplement: Supplementary file 3 — Reporting Summary [file 41467_2019_13935_MOESM3_ESM.pdf]

nature research

Corresponding author(s): DBPR

Last updated by author(s): Nov 30, 2019

Reporting Summary

Nature Research wishes to improve the reproducibility of the work that we publish. This form provides structure for consistency and transparency in reporting. For further information on Nature Research policies, see Authors & Referees and the Editorial Policy Checklist.

Statistics

For all statistical analyses, confirm that the following items are present in the figure legend, table legend, main text, or Methods section.

n/a	Confirmed	
☐	☒	The exact sample size (n) for each experimental group/condition, given as a discrete number and unit of measurement
☒	☐	A statement on whether measurements were taken from distinct samples or whether the same sample was measured repeatedly
☐	☒	The statistical test(s) used AND whether they are one- or two-sided<br>*Only common tests should be described solely by name; describe more complex techniques in the Methods section.*
☐	☒	A description of all covariates tested
☐	☒	A description of any assumptions or corrections, such as tests of normality and adjustment for multiple comparisons
☐	☒	A full description of the statistical parameters including central tendency (e.g. means) or other basic estimates (e.g. regression coefficient) AND variation (e.g. standard deviation) or associated estimates of uncertainty (e.g. confidence intervals)
☐	☒	For null hypothesis testing, the test statistic (e.g. F, t, r) with confidence intervals, effect sizes, degrees of freedom and P value noted<br>*Give P values as exact values whenever suitable.*
☒	☐	For Bayesian analysis, information on the choice of priors and Markov chain Monte Carlo settings
☒	☐	For hierarchical and complex designs, identification of the appropriate level for tests and full reporting of outcomes
☒	☐	Estimates of effect sizes (e.g. Cohen's d, Pearson's r), indicating how they were calculated

Our web collection on statistics for biologists contains articles on many of the points above.

Software and code

Policy information about availability of computer code

Data collection	Geolocated tweets were collected using the Twitter API. Tide gauge, weather and population data were collected from publicly-available online sources as documented in Methods.
Data analysis	Analysis was performed using R v.3.6.1

For manuscripts utilizing custom algorithms or software that are central to the research but not yet described in published literature, software must be made available to editors/reviewers. We strongly encourage code deposition in a community repository (e.g. GitHub). See the Nature Research guidelines for submitting code & software for further information.

Data

Policy information about availability of data

All manuscripts must include a data availability statement. This statement should provide the following information, where applicable:

- Accession codes, unique identifiers, or web links for publicly available datasets
- A list of figures that have associated raw data
- A description of any restrictions on data availability

Source data to generate all manuscript figures is provided with the manuscript. Tide gauge data is from https://tidesandcurrents.noaa.gov/ . Precipitation data is from PRISM http://www.prism.oregonstate.edu/ . We collected our Twitter data from the public domain in adherence with Twitter's Developer Agreement. Twitter data are restricted from public redistribution by the Twitter terms of service. Raw Twitter data may be procured through Twitter's GNIP service.

Field-specific reporting

Please select the one below that is the best fit for your research. If you are not sure, read the appropriate sections before making your selection.

☐ Life sciences ☒ Behavioural & social sciences ☐ Ecological, evolutionary & environmental sciences

For a reference copy of the document with all sections, see nature.com/documents/nr-reporting-summary-flat.pdf

Behavioural & social sciences study design

All studies must disclose on these points even when the disclosure is negative.

Study description	Quantitative panel data and time-series analysis
Research sample	Twitter users in shoreline counties along the east and Gulf coast of the United States
Sampling strategy	Tweets geolocated within shoreline counties
Data collection	Tweets were collected using the Twitter API between March 2014 and November 2016. Tide gauge, weather and population data were obtained from public online sources as documented in Methods.
Timing	March 2014 toNovember 2016
Data exclusions	Counties with flood-related tweets on fewer than 20% of days were excluded from the county-specific analysis.
Non-participation	NA
Randomization	NA

Reporting for specific materials, systems and methods

We require information from authors about some types of materials, experimental systems and methods used in many studies. Here, indicate whether each material, system or method listed is relevant to your study. If you are not sure if a list item applies to your research, read the appropriate section before selecting a response.

Materials & experimental systems

n/a	Involved in the study	
☒	☐	Antibodies
☒	☐	Eukaryotic cell lines
☒	☐	Palaeontology
☒	☐	Animals and other organisms
☒	☐	Human research participants
☒	☐	Clinical data

Methods

n/a	Involved in the study	
☒	☐	ChIP-seq
☒	☐	Flow cytometry
☒	☐	MRI-based neuroimaging
